# RTMS Mitigates Simulated Spaceflight‐Induced Emotional Dysfunction Through VEGFR‐2‐Mediated Neuroprotection

**DOI:** 10.1002/brb3.71799

**Published:** 2026-09-28

**Authors:** Rong Liang, Qing Xu, Jing Ren, Ling Wang, Xinyan Cheng, Fei Zhao, Jing Xu, Jiajia Yang, Dong Ming

**Affiliations:** ^1^ Medical School, Faculty of Medicine Tianjin University Tianjin China; ^2^ Haihe Laboratory of Brain‐Computer Interaction and Human‐Machine Integration Tianjin China; ^3^ Tianjin Medical University General Hospital Tianjin China; ^4^ State Key Laboratory of Advanced Medical Materials and Devices Tianjin University Tianjin China

**Keywords:** emotion, neuroprotection, repetitive transcranial magnetic stimulation, simulated space composite environment, VEGFR‐2

## Abstract

**Purpose:**

Repetitive transcranial magnetic stimulation (rTMS) has shown potential in mitigating emotional deficits induced by extreme environments, such as those encountered during spaceflight. However, its underlying molecular mechanisms remain poorly understood. This study investigates the role of vascular endothelial growth factor receptor 2 (VEGFR‐2) in mediating the therapeutic effects of rTMS in a mouse model of simulated space composite environment (SSCE).

**Method:**

10 Hz rTMS treatment was initiated after the last two weeks of SSCE exposure and continued for the remaining 2 weeks. Throughout the rTMS treatment period, the VEGF receptor antagonist SU5416 was co‐administered via intraperitoneal injection.

**Finding:**

Behavioral tests revealed that VEGFR‐2 inhibition abolished the beneficial effects of rTMS on depressive‐ and anxiety‐like behaviors, as measured by the tail suspension, forced swim, sucrose preference, and elevated plus maze tests. Furthermore, SU5416 blocked rTMS‐induced improvement in social skills. At the cellular level, VEGFR‐2 antagonism impaired axonal integrity in the prefrontal cortex.

**Conclusion:**

These findings suggest that VEGFR‐2 signaling is critical for the neuroprotective and behavioral benefits of rTMS under simulated spaceflight conditions, highlighting its potential as a therapeutic target for emotional regulation in extreme environments.

## Graphical abstract

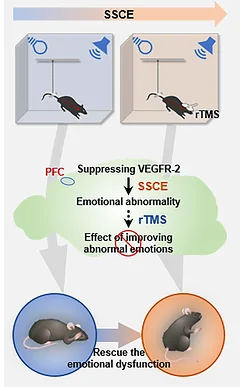

## 1 Introduction

Human spaceflight missions aboard space stations are becoming increasingly frequent, representing merely the initial step in exploring the vast universe. It is anticipated that more ambitious objectives, such as manned missions to Mars, may be achieved in the near future (Romanella et al., 2020). During spaceflight, astronauts themselves constitute the most critical determinant of mission success, as their ability to maintain high‐level performance directly impacts operational outcomes. Aboard space stations, astronauts are exposed to various stressors, including microgravity, confinement, isolation, noise, and circadian rhythm disruption (Oluwafemi et al., 2021; Desai et al., 2022; Scott et al., 2022). These complex environmental conditions not only impair physiological functions but may also contribute to adverse psychological states such as anxiety and depression (Shang et al., 2017; Wang et al., 2018). Previous studies have demonstrated that emotional disturbances can emerge after just 1 week of exposure to a simulated extreme environment and may persist with prolonged duration (Xu et al., 2024).

Repetitive transcranial magnetic stimulation (rTMS) is a non‐invasive brain stimulation technique based on the principle of electromagnetic induction (Bai et al., 2023). It uses pulsed currents to generate dynamic magnetic fields that induce transient neuronal excitation and can exert long‐term effects both in specific cortical regions and deeper brain structures (Platz and Rothwell, 2010). The changes induced by rTMS are not limited to the stimulated area and may not be temporary; they can also affect functionally connected distal regions, thereby facilitating long‐term modulation of brain plasticity. Potential mechanisms include the regulation of neurotrophic factors and neurotransmitter systems (Baeken et al., 2011; Wang et al., 2011; Wang et al., 2015). Vascular endothelial growth factor (VEGF), a key mediator of angiogenesis and vascular permeability, is an important neurotrophic and angiogenic factor (Lange et al., 2016; Pu et al., 2020; Tseng et al., 2015). VEGF‐A may contribute to the reconstruction of the neurovascular unit (Udo et al., 2008). Its neuroprotective effects include stimulating axonal growth, protecting cells from apoptosis, and promoting neurogenesis in the subventricular zone and hippocampus (Hecking et al., 2022; Gao et al., 2013). The presence and significance of VEGF in the central nervous system are well‐established. Accumulating evidence suggests that VEGF and its receptor VEGFR‐2 mediate key neurobiological processes involved in antidepressant treatment responses (Newton et al., 2013; Clark‐Raymond and Halaris, 2013).

Studies have shown that rTMS improves learning and memory in vascular dementia model rats by upregulating the expression of VEGF, BDNF, and NMDARs (Zhang et al., 2015). Refractory depression patients who achieved remission after rTMS treatment exhibited increased serum VEGF levels (Fukuda et al., 2020). These findings indicate that VEGF binding to VEGFR‐1 and VEGFR‐2 (Ruiz de Almodovar et al., 2009) in the mammalian brain is involved in regulating neurogenesis (Jin et al., 2002) and may at least partially mediate the antidepressant effects of both conventional antidepressants (Warner‐Schmidt and Duman, 2007) and ketamine (Deyama et al., 2019; Choi et al., 2016). Previous work has demonstrated that 2 weeks of 10 Hz rTMS application ameliorates emotional abnormalities in mice exposed to a simulated space composite environment (SSCE) (Liang et al., 2023, Liang et al., 2025), accompanied by increased VEGFR‐2 expression in the prefrontal cortex (Xu et al., 2024), suggesting that VEGF signaling may contribute to the observed behavioral improvements. However, whether this signaling pathway is required for rTMS efficacy remains to be determined. Here, we move beyond correlative observations by pharmacologically blocking VEGFR‐2 with SU5416 to test the functional involvement of this receptor for the neuroprotective and behavioral effects of rTMS. We further investigate whether VEGFR‐2 inhibition disrupts rTMS‐mediated preservation of neuronal integrity, thereby providing further mechanistic insight into the antidepressant and anxiolytic actions of rTMS in extreme environments.

## 2 Material and Methods

### 2.1 Animals

Male C57BL/6J mice aged 6–8 weeks were used in the experiments. Mice were housed at 22 ± 2°C with a humidity of 50 ± 10% under a 12‐h light‐dark cycle. All husbandry and experimental procedures in this study were approved by the Animal Care and Use Committee of Tianjin University. Throughout the experiment, the health of the mice was closely monitored, including parameters such as body weight, fur condition, and the presence of wounds. Mice displaying any signs of health abnormalities were removed from the study.

### 2.2 Simulated Space Composite Environment Exposure

The SSCE mouse model was an enhanced version of previous models (Xu et al., 2024; Liang et al., 2023; Morey‐Holton and Globus, 2002). In this setup, 30° tail suspension was used to mimic microgravity. Mice were placed in a restrainer, with only their tails exposed. Their tails were washed with warm water, dried with paper towels, and then wrapped in medical gauze. One end of a nylon string was secured to the tail using medical tape and breathable gauze, while the other end was attached to a keyring suspended from the crossbeam of a 23 × 30 × 18 cm^3^ cubic cage. By adjusting the length of the nylon string, the mouse's body was positioned at a 30° angle relative to the ground. The keyring allowed the mice to rotate freely in a horizontal plane (360°), and they had continuous access to food and water. The composite environment also included social isolation (housing the mice individually), confinement to a small space, exposure to the real background noise of “Tiangong‐2” (60‐75 dB), and a 90‐minute light‐dark cycle. The total duration of exposure in this model was 4 weeks.

### 2.3 Repetitive Transcranial Magnetic Stimulation

From the third week of modeling, whole‐brain rTMS was initiated, and mice were placed in a custom‐made mouse fixator, which was a 50‐mL centrifuge tube with uniform ventilation holes, allowing breathing and free rotation horizontally but not vertically. Magnetic stimulation was performed using a stimulator from the Institute of Electrical Engineering, Chinese Academy of Sciences, with a coil diameter of approximately 7.5 cm. The stimulation parameters were set to 10 Hz frequency, 1 T intensity, with each sequence consisting of 100 pulses, and a 20‐second interval between trains of 10 pulses (Tian et al., 2020; Hou et al., 2021). Stimulation was administered daily at 5:00 p.m. for 14 consecutive days. After 4 weeks of modeling and rTMS treatment, the gauze was carefully removed for subsequent experiments. The control group was housed in standard cages with 4–5 animals per cage under normal conditions.

### 2.4 SU5416 Administration

Mice in the SSCE + rTMS + SU5416 group received intraperitoneal (i.p.) injections of SU5416 (20 mg/kg) (Castañeda‐Cabral et al., 2023), commencing in the third week of the modeling period. Injections were administered every other day at 10:00 AM for two weeks, until the end of the modeling protocol. The drug was dissolved in a vehicle consisting of 5% DMSO, 40% PEG300, 5% Tween‐80, and 50% saline. Body weight was measured prior to each injection to adjust the dosage accordingly. Concurrently, control (CON) and SSCE + rTMS group mice received i.p. injections of an equal volume of the vehicle solution without SU5416. The final injection was conducted separately from the behavioral tests.

### 2.5 Method Details

#### 2.5.1 Western Blot Experiment

As previously described (Sun et al., 2017; Dang et al., 2022), PFC tissue was homogenized and lysed on ice for 30 min using RIPA buffer. The lysate was then centrifuged at 12,000 rpm for 20 min (Eppendorf, Germany), and the supernatant was collected. Protein concentration was determined using the BCA assay kit (Solarbio, China). A total of 20 µg of protein was separated by SDS‐PAGE at 120 V for 90 min, followed by transfer to a PVDF membrane at 100 V for 90 min. The membrane was blocked with 5% skim milk for 60 min. The membranes were incubated at 4°C overnight with GAPDH (1:1000, GeneTex, GTX100118) and VEGFR‐2 (1:1000, Proteintech, 26415‐1‐AP). After washing with TBST (3 × 10 min), membranes were incubated with HRP‐conjugated secondary antibodies (rabbit IgG or mouse IgG (HRP), 1:2000) for 40 min, followed by TBST washing (3 × 10 min). GAPDH was used as the internal control. Protein bands were visualized using an automatic imaging system (Tanon, China).

#### 2.5.2 Histology and Immunohistochemistry

After cardiac perfusion with PBS, the whole brain was collected. Brain tissue was fixed overnight in 4% paraformaldehyde. The tissue was then dehydrated sequentially with 20%, 30%, and 30% sucrose solutions until the tissue settled at the bottom of the centrifuge tube. Coronal sections (30 µm thick) were cut using a frozen slicer (Leica, Germany) at −20°C. The sections were washed three times for 3 min each with 0.1 M PBS and PBS‐T. Blocking was performed for 1 h in 0.1 M PBS containing 10% BSA (Solarbio, China) and 0.5% Triton X‐100 (DINGGUO CHANGSHENG BIOTECHNOLOGY CO. LTD., China). The sections were incubated overnight at 4°C with VEGFR‐2 (1:200, Proteintech, 26415‐1‐AP), NeuN (1:5000, Proteintech, 66836‐1‐Ig), and SMI312 (1:250, Biolegend, 837904). The following day, after washing with PBS‐T (3 × 3 min), the sections were incubated for 1 h with either Alexa Fluor 488‐labeled goat anti‐rabbit IgG (H + L) antibody (1:500, Beyotime) or Alexa Fluor 555‐labeled donkey anti‐mouse IgG (H + L) antibody (1:500, Beyotime). The sections were then washed with 0.1 M PBS (3 × 3 min). Finally, the sections were mounted and stained with DAPI (Beyotime, China) for immunofluorescence imaging. Images were acquired using a confocal laser scanning microscope (Nikon, Japan).

### 2.6 Behavioral Assays

#### 2.6.1 Tail Suspension Test

The tail suspension test (TST) is used to assess depressive‐like behavior in mice (Cryan et al., 2005). The mouse's tail is affixed to a metal rod with tape, and the animal is suspended vertically. Its behavior is recorded in real‐time, with the duration of both struggle and immobility analyzed over a 5‐min period.

#### 2.6.2 Forced Swim Test

The forced swim test (FST) is another classic method for evaluating depressive‐like behavior (Bogdanova et al., 2013). It is conducted in a cylindrical container (15 cm in diameter, 30 cm in height) filled with water to a depth of 15 cm at a temperature of 23 ± 2°C. The mouse's behavior is continuously recorded, with the duration of both struggle and immobility within a 5‐minute period being analyzed. Immobility is defined as the period during which the mouse makes only minimal movements to keep its head above water.

#### 2.6.3 Sucrose Preference Test

The sucrose preference test (SPT) is used to assess anhedonic behavior in mice (Liu et al., 2018). During the initial phase, mice were acclimated to two bottles of 1% sucrose solution for 24 h. In the subsequent phase, one bottle of sucrose was replaced with tap water for another 24‐h adaptation period. Following this, the animals underwent 24 h of food and water deprivation. In the test phase, mice were placed in their home cages with access to one bottle of 1% sucrose solution and one bottle of water. Sucrose and water consumption were recorded over a 2‐h period, and a sucrose preference index was calculated as follows: Preference Index = [Sucrose intake/(Sucrose intake + Water intake)] × 100.

#### 2.6.4 Elevated Plus Maze Test

The elevated plus‐maze (EPM) test is used to assess anxiety‐like behavior in mice (Komada et al., 2008). It consists of two open arms (30 × 7 cm^2^) and two enclosed arms (30 × 7 × 20 cm^3^), arranged perpendicularly at the center (7 × 7 cm^2^). The apparatus is positioned about one meter off the ground. Mice are placed at the center, facing the open arms. The environment is kept dark and quiet to minimize external variables that could influence the results. Mice are allowed to freely explore for 5 min while their movement trajectories and behavioral metrics are recorded via video tracking software. After each trial, the maze is cleaned with 75% alcohol and water to prevent contamination between trials.

#### 2.6.5 Three‐Chamber Social Interaction Test

The three‐chamber social interaction apparatus consists of an acrylic cube (60 × 40 × 30 cm^3^) divided into three equal sections (Figure 3a). The two outer sections contain inverted cylindrical containers (referred to as Empty 1 and Empty 2). During adaptation, the test mouse is placed in the central chamber and allowed to explore freely for 5 min. In Stage 1, a stranger mouse (Stranger 1), age‐ and sex‐matched to the test mouse, is placed in one of the empty chambers, and the test mouse is returned to the central chamber for another 5 min of free exploration. The chamber with Stranger 1 is designated as the social zone, the other empty chamber as the non‐social zone, and the central chamber as the neutral zone. In Stage 2, another stranger mouse (Stranger 2), age‐ and sex‐matched to the test mouse, is placed in the second empty chamber, and the test mouse is allowed to explore for 5 min. After each trial, the apparatus is cleaned with 75% alcohol followed by water to prevent contamination in subsequent experiments.

### 2.7 Quantification and Statistical Analysis

Statistical analyses were conducted using Origin 2021 (OriginLab Corporation, United States) and SPSS Statistics 20 (IBM, United States). Sample sizes were not predetermined using statistical methods but were consistent with those reported in previous studies. For behavioral tests, the sample sizes were CON *n* = 6, SSCE + rTMS *n* = 6, and SSCE + rTMS + SU5416 *n* = 5 (one mouse in the SU5416 group was excluded due to tail injury during SSCE exposure). For Western blot analyses, n = 4 mice per group were used; for immunofluorescence, 3 sections per mouse were quantified, and the average value per animal was used for statistical analysis, with the animal as the unit of analysis. Data collection and analysis were performed under experimental conditions, with the number of animals (*n*) provided in the figure legends. Given the small sample sizes (*n* = 3–6 per group), the Shapiro–Wilk test has limited power; therefore, normality was assessed primarily by visual inspection of Q‐Q plots, with Shapiro–Wilk results used as supplementary information. Homogeneity of variance was examined using Levene's test. One‐way analysis of variance (ANOVA) was used for comparisons of behavioral, Western blot, and immunofluorescence data among three groups, followed by LSD post hoc tests for equal variances or Tamhane's T2 test for unequal variances. For the three‐chamber social interaction test, two‐way repeated‐measures ANOVA was applied at each stage, with group as the between‐subjects factor and chamber type as the within‐subjects factor; post hoc comparisons were performed when significant interactions were detected. All reported p‐values are from post hoc tests. Data are presented as group means ± S.E.M. (***p < 0.001, **p < 0.01, and *p < 0.05).

## 3 Results

### 3.1 Suppressing VEGFR‐2 Blocks rTMS‐modulated Improvement of Emotional Deficits in SSCE Exposure

Although we have previously demonstrated that rTMS ameliorated emotional abnormalities induced by SSCE exposure by enhancing the activity of underactive mPFC neurons (Liang et al., 2023), the underlying mechanisms remain unclear. VEGF has been identified as a key molecule linking neuroplasticity and emotional behavior (Sun et al., 2016; Zhao et al., 2010). To investigate the role of VEGF in this process, mice were exposed to SSCE for four weeks (Figures 1a, c). During the latter two weeks, rTMS was administered concomitantly with intraperitoneal injection of the VEGFR‐2 inhibitor SU5416 (Figure 1b). SU5416 significantly suppressed VEGFR‐2 protein expression in the prefrontal cortex (Figures 1d, e, one‐way ANOVA, *F*_(2, 9)_ = 4.622, SSCE + rTMS vs. SSCE + rTMS + SU5416: *p* < 0.05). Behaviorally, compared with the control and SSCE + rTMS groups, VEGFR‐2 inhibition (as assessed in the PFC) markedly increased immobility time in both the TST (Figures 2a, b, one‐way ANOVA, *F*_(2, 14)_ = 7.389, CON vs. SSCE + rTMS + SU5416: *p* < 0.01; SSCE + rTMS vs. SSCE + rTMS + SU5416: *p* < 0.05) and FST (Figures 2c, d, one‐way ANOVA, *F*_(2, 14)_ = 6.716, CON vs. SSCE + rTMS + SU5416: *p* < 0.01; SSCE + rTMS vs. SSCE + rTMS + SU5416: *p* < 0.05), and reduced the sucrose preference index in the SPT (Figures 2e, f, one‐way ANOVA, *F*_(2, 14)_ = 6.496, CON vs. SSCE + rTMS + SU5416: *p* < 0.01; SSCE + rTMS vs. SSCE + rTMS + SU5416: *p* < 0.01). These results suggested that blocking VEGFR‐2 abolished the protective effects of rTMS against depressive‐like behaviors following SSCE exposure. Furthermore, in the elevated plus maze test—a measure of anxiety‐like behavior—mice with VEGFR‐2 inhibition spent significantly less time in the open arms (Figures 2g, h, one‐way ANOVA, *F*_(2, 14)_ = 8.970, CON vs. SSCE + rTMS + SU5416: *p* < 0.01; SSCE + rTMS vs. SSCE + rTMS + SU5416: *p* < 0.05) and entered the open arms less frequently (Figure 2i, one‐way ANOVA, *F*_(2, 14)_ = 9.391, CON vs. SSCE+rTMS: *p* < 0.05; CON vs. SSCE + rTMS + SU5416: *p* < 0.01) compared to controls, independent of general locomotor activity (Figure 2j, one‐way ANOVA, *F*_(2, 14)_ = 3.364, CON vs. SSCE + rTMS + SU5416: *p*>0.05; SSCE + rTMS vs. SSCE + rTMS + SU5416: *p* > 0.05). Together, these findings indicated that suppression of VEGFR‐2 signaling impeded the beneficial effects of rTMS on both depressive‐like and anxiety‐like behaviors induced by SSCE.

**FIGURE 1:**
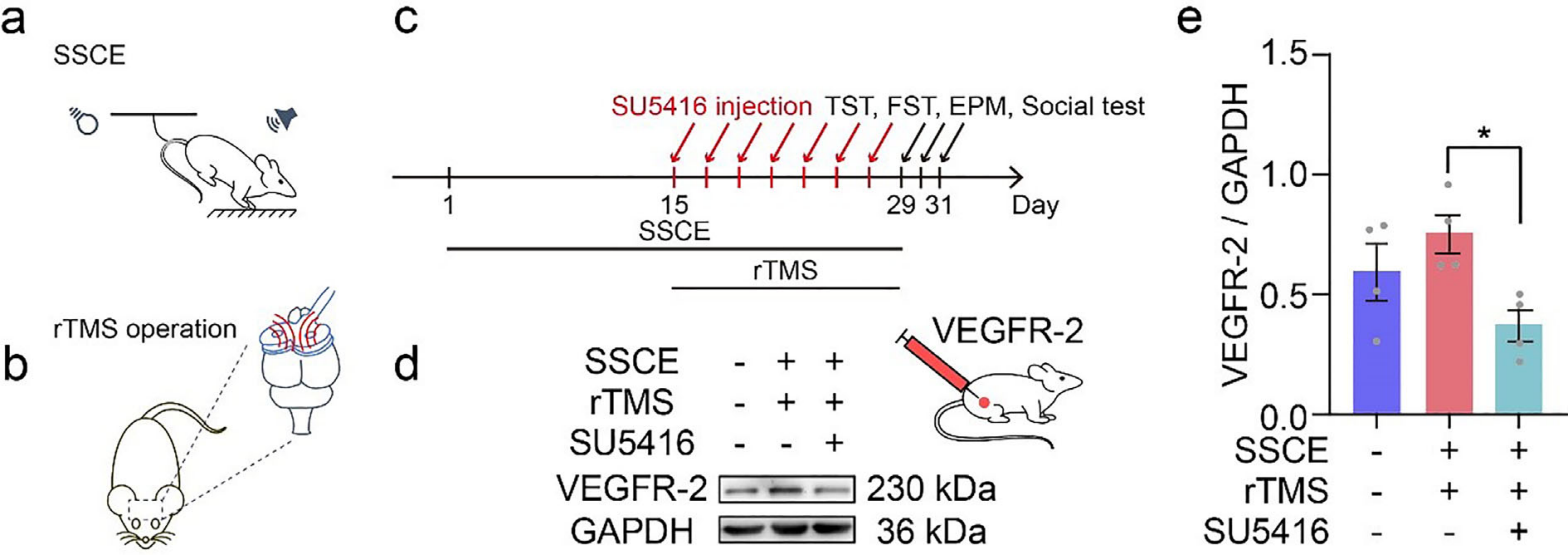
SU5416 inhibited the expression of VEGFR‐2 in the prefrontal cortex. a, b. Schematic diagram of SSCE (a) and rTMS (b) application. (c) Schematic of the paradigm. (d) Representative western blot bands using total protein extracts from the PFC region. (e) Quantification of protein expression levels in the PFC among the CON, SSCE + rTMS, and SSCE + rTMS + SU5416 groups (from four mice in each group).

**FIGURE 2:**
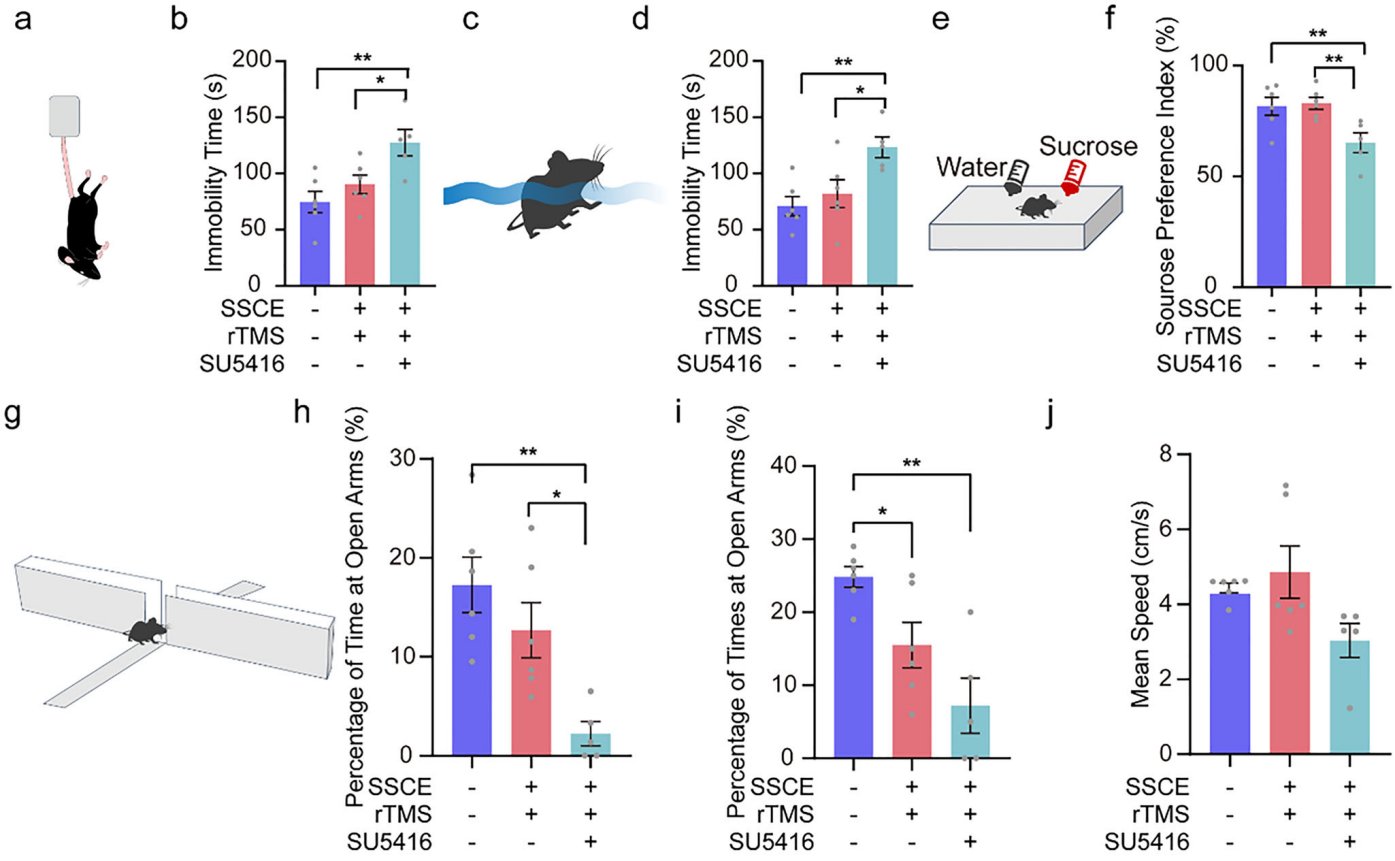
SU5416 blocked the improvement effect of rTMS on the emotional behavior of SSCE mice. a, b. Schematic diagrams (a) and statistical results (b) of the time spent immobile within 5 min in the TST for each group of mice (CON: *n* = 6 mice, SSCE + rTMS: *n* = 6 mice, SSCE + rTMS + SU5416: *n* = 5 mice). c, d. Schematic diagrams (c) and statistical results (d) of the time spent immobile within 5 min in the FST for each group of mice (CON: *n* = 6 mice, SSCE + rTMS: *n* = 6 mice, SSCE + rTMS + SU5416: *n* = 5 mice). e, f. Schematic diagrams (e) and statistical results (f) of the percentage of sucrose preference coefficient of each group of mice within 2 h in the SPT for each group of mice (CON: *n* = 6 mice, SSCE + rTMS: *n* = 6 mice, SSCE + rTMS + SU5416: *n* = 5 mice). g‐i. Schematic diagrams (g) and statistical results of the percentage of time (h) and frequency (i) of open arm stay of each group of mice within 5 min in the EPM for each group of mice (CON: *n* = 6 mice, SSCE + rTMS: *n* = 6 mice, SSCE + rTMS + SU5416: *n* = 5 mice). (j) The average walking speed of mice in each group in the EPM.

### 3.2 Suppressing VEGFR‐2 Blocks rTMS‐Modulated Improvement of Social Skills Deficits Induced by SSCE Exposure

Furthermore, we examined the effect of inhibiting VEGFR‐2 expression on social behavior in mice using the classic Three‐Chamber Social test, which assesses social preference and social novelty preference (Figure 3a). During the habituation phase, no significant position preference was observed in the experimental mice (Figure 3b, two‐way repeated ANOVA, *p>*0.05), ensuring an unbiased baseline for subsequent tests. After a 5‐min acclimation period, an unfamiliar mouse (Stranger 1), matched in age and sex, was placed under an inverted wire cup in one of the chambers. Consistent with the natural tendency of mice to interact more with a conspecific than with an empty cup, control animals and those in the SSCE + rTMS group showed increased sniffing time toward Stranger 1 (Figure 3c, two‐way repeated ANOVA, CON‐Stranger 1 vs. CON‐Empty: *p* < 0.001; SSCE + rTMS‐Stranger 1 vs. SSCE + rTMS‐Empty: *p* < 0.01). However, VEGFR‐2 inhibition (with PFC as the sampled region) significantly reduced the sniffing time directed toward Stranger 1 compared to both control and SSCE + rTMS groups (two‐way repeated ANOVA, Stranger 1: CON vs. SSCE + rTMS + SU5416: *p* < 0.01; SSCE + rTMS vs. SSCE + rTMS + SU5416: *p* < 0.001).

**FIGURE 3:**
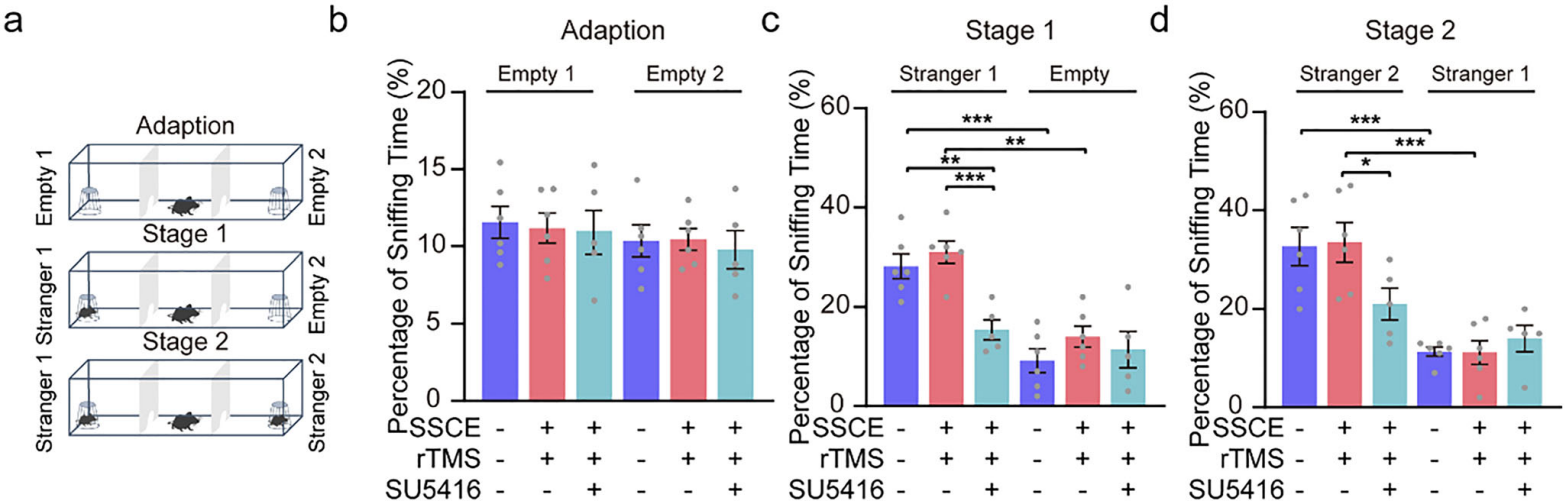
SU5416 blocked the improvement effect of rTMS on the social behavior of SSCE mice. a. Schematic diagrams of three‐chamber social tests. b‐d. The percentages of sniffing time for the test mice at different stages (b: Adaption, c: Stage 1, d: Stage 2), respectively (CON: *n* = 6 mice, SSCE + rTMS: *n* = 6 mice, SSCE + rTMS + SU5416: *n* = 5 mice).

In the second phase, another unfamiliar mouse (Stranger 2), also matched in age and sex, was introduced into a previously empty cup to evaluate social novelty preference. The experimental mice generally preferred sniffing the novel Stranger 2 over the familiar Stranger 1 (Figure 3d, two‐way repeated ANOVA, CON‐Stranger 2 vs. CON‐Stranger 1: *p* < 0.001; SSCE + rTMS‐Stranger 2 vs. SSCE + rTMS‐Stranger 1: *p* < 0.001). For social novelty recognition (Stranger 2), sniffing time was significantly reduced in the SSCE + rTMS + SU5416 group compared with the SSCE + rTMS group (two‐way repeated ANOVA, Stranger 2: SSCE + rTMS vs. SSCE + rTMS + SU5416: *p* < 0.05). These results indicated that suppression of VEGFR‐2 in the PFC impaired the beneficial effect of rTMS on SSCE‐induced deficits in social skills.

### 3.3 VEGFR‐2 Inhibition Blocks the Neuroprotective Benefits of rTMS Against SSCE‐Induced Deficits

Previous studies have suggested that the impact of VEGF on emotional behavior may be related to its neuroprotective effects (Abd Al Haleem et al., 2021), which could explain why inhibition of VEGFR‐2 expression blocks the improvement in emotional behavior induced by rTMS under SSCE exposure. Therefore, we used immunofluorescence staining to first examine the distribution of VEGFR‐2 in the PFC (Figure 4a). We found that administration of SU5416 significantly suppressed VEGFR‐2 expression in the PFC compared to the CON and SSCE + rTMS groups (Figure 4b, one‐way ANOVA, *F*_(2, 6)_ = 8.056, CON vs. SSCE + rTMS + SU5416: *p* < 0.01; SSCE + rTMS vs. SSCE + rTMS + SU5416: *p* < 0.05), consistent with quantitative protein expression results. We also observed the number of mature neurons and the distribution of axons in the PFC across the three groups (Figures 4c, e). VEGFR‐2 inhibition (with PFC as the sampled region) showed a decreasing trend in the number of mature neurons compared to the SSCE + rTMS group, although this reduction did not reach statistical significance (Figure 4d: one‐way ANOVA, *F*_(2, 6)_ = 2.625, CON vs. SSCE + rTMS + SU5416: *p* > 0.05). In contrast, VEGFR‐2 inhibition significantly reduced axonal integrity compared to the control group (Figure 4f: one‐way ANOVA, *F*_(2, 6)_ = 29.367, CON vs. SSCE + rTMS + SU5416: *p* < 0.001). Moreover, mice in the SSCE + rTMS + SU5416 group showed marked reductions in SMI‐312 staining signals in the PFC compared to the SSCE + rTMS group (Figure 4f: SSCE + rTMS vs. SSCE + rTMS + SU5416: *p* < 0.01), indicating axonal degeneration following SU5416 administration. These findings indirectly demonstrated the neuroprotective role of VEGF.

**FIGURE 4:**
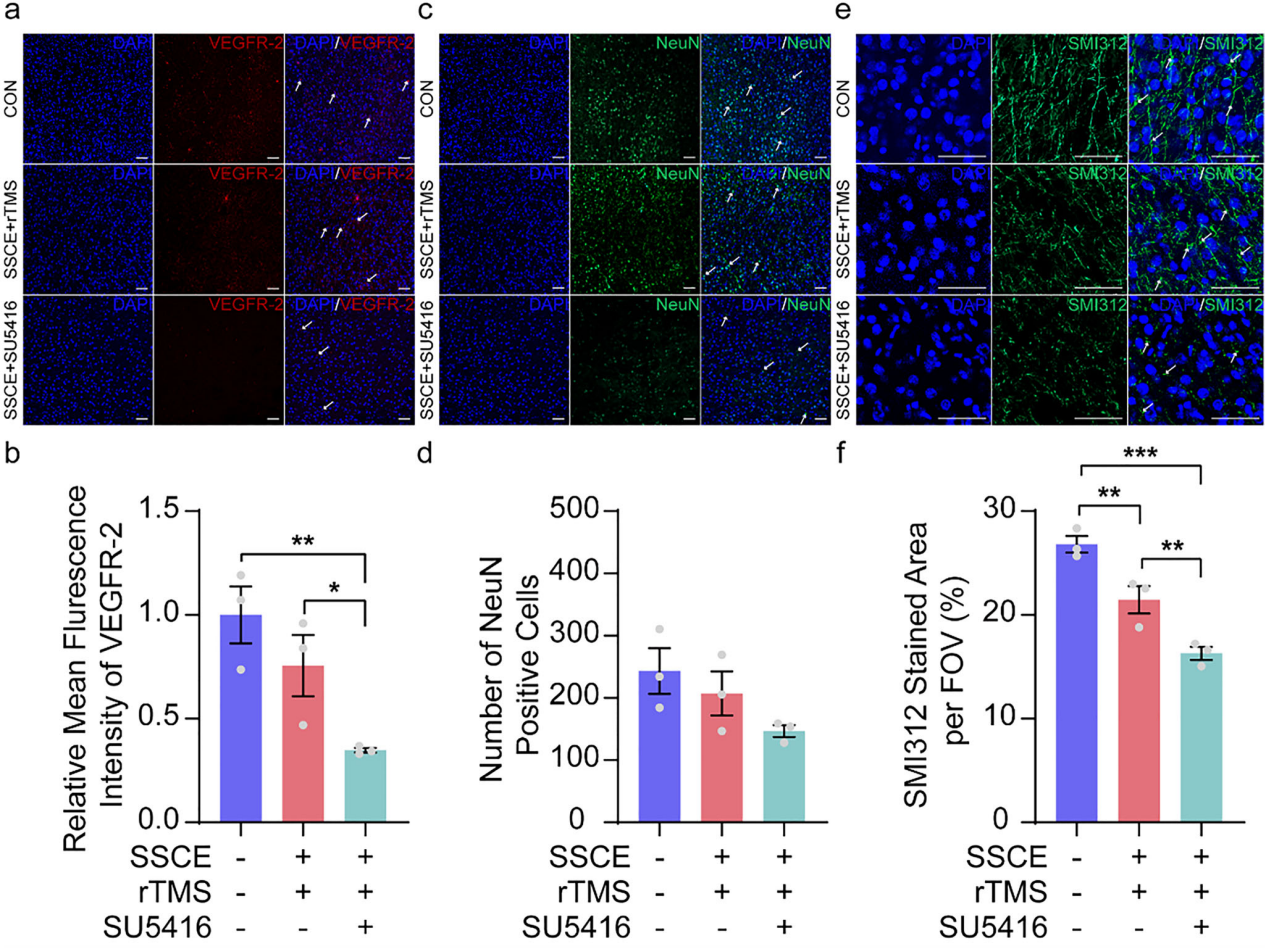
Verification of the expression of molecules related to neuroprotection. a, b. Representative images (a) and statistical results (b) showing the VEGFR‐2 immunofluorescence, respectively, scale bar, 50 µm, *n* = 3 mice. c, d. Representative images (c) and statistical results (d) showing the NeuN immunofluorescence, respectively, scale bar, 50 µm, *n* = 3 mice. e, f. Representative images (e) and statistical results (f) showing the SMI312 immunofluorescence, respectively, scale bar, 50 µm, *n* = 3 mice.

## 4 Discussion

Unlike NASA's Hindlimb Unloading (HLU) model, which focuses mainly on physiological changes (Willey et al., 2022), our four‑week composite paradigm simultaneously incorporates multiple spaceflight stressors—30° tail‑suspension, social isolation, 60–75 dB cabin noise, and 90‑min light/dark cycles—to better mirror real mission conditions. This integrated design allows us to explore how varying exposure durations influence prefrontal cortical molecular networks underlying emotional regulation. The ameliorative effects of rTMS on emotional abnormalities in mice under SSCE have been previously established, and it has been confirmed that its regulatory role may be associated with enhanced activity of hypoactive excitatory neurons in the medial prefrontal cortex (Liang et al., 2023; Liang et al., 2022). In this study, we focused on investigating the molecular mechanisms through which 10 Hz rTMS improves emotional function. rTMS treatment was initiated after the last 2 weeks of SSCE exposure and continued for the remaining 2 weeks. Throughout the rTMS treatment period, the VEGF receptor antagonist SU5416 was co‐administered via intraperitoneal injection. Inhibition of VEGFR‐2 expression was found to counteract the beneficial effects of rTMS on anxiety‐ and depression‐like behaviors in SSCE‐exposed mice, as assessed in the tail suspension, forced swim, sucrose preference, and elevated plus maze tests. In the social interaction test, VEGFR‐2 inhibition blocked the rTMS‐mediated improvement in social preference impairment induced by SSCE, though it had no significant effect on social novelty behavior. Furthermore, immunofluorescence staining suggested that the alterations in emotional behavior may be related to the disruption of rTMS‐induced neuroprotection by VEGF inhibition. These results demonstrate that the applied rTMS protocol is effective at mitigating emotional disturbances under SSCE conditions, although further studies in both animal models and humans are necessary to refine and validate this therapeutic strategy. Notably, whereas our 2024 study (Xu et al., 2024) demonstrated an association between rTMS, VEGF upregulation, and behavioral recovery, the present work provides evidence that VEGFR‐2 signaling plays a critical role in these effects. By showing that SU5416 abolishes rTMS‐induced improvements in depressive‐ and anxiety‐like behaviors and simultaneously reduces mature neuron counts and axonal integrity in the prefrontal cortex, we identify VEGFR‐2 as a key molecular effector linking rTMS to neuroprotection. Although rTMS and SU5416 were delivered globally, the PFC was chosen as the primary region of analysis given its well‐established role in emotional and social behavior regulation. Our findings therefore highlight the PFC as a key locus of VEGFR‐2‐mediated effects, without excluding potential contributions from other brain regions. This represents a significant mechanistic advance by identifying a specific receptor and a downstream cellular pathway that are essential for rTMS therapeutic efficacy under simulated spaceflight conditions.

The present findings underscore the critical involvement of VEGF/VEGFR‐2 signaling in mediating the beneficial behavioral and neuroplastic effects of rTMS under simulated spaceflight conditions. Our data align with and extend previous reports indicating that VEGF not only promotes angiogenesis but also exerts significant neurotrophic and neuroprotective functions within the central nervous system (Le et al., 2021; Rosenstein et al., 2020). Specifically, the abolition of rTMS‐induced improvements in emotional and social behaviors following VEGFR‐2 antagonism suggests that VEGF signaling is integral to the mechanism through which rTMS counteracts SSCE‐induced deficits. This is consistent with studies demonstrating that VEGF and its receptors are implicated in the pathophysiology of mood disorders and the mechanisms of antidepressant treatments (Matsuno et al., 2022; Deyama et al., 2023; Sideromenos et al., 2020). For instance, Deyama et al. highlighted the essential role of VEGF signaling in the prefrontal cortex for the rapid antidepressant effects of ketamine (2019), while Zhang et al. showed that rTMS upregulates VEGF and facilitates synaptic plasticity in a model of vascular dementia (2015). VEGFR‐2 antagonism abolishes antidepressant‐like behavioral effects in preclinical models of depression (Warner‐Schmidt and Duman, 2007; Greene et al., 2009). Previous studies have also shown that infusion of BDNF or VEGF antibodies into the mPFC blocks the antidepressant effects of Resolvin E1 (Deyama et al., 2023). Our results further substantiate the notion that VEGF‐VEGFR‐2 activation may represent a common pathway underpinning the therapeutic effects of diverse neuromodulatory interventions.

At the cellular level, our immunofluorescence data provide compelling evidence that the behavioral effects of VEGFR‐2 inhibition are paralleled by reductions in mature neuron counts and axonal integrity within the PFC. This aligns with established roles of VEGF in promoting neuronal survival, neurogenesis, and axonal outgrowth (Carmeliet and de Almodovar, 2013; Ribatti and Guidolin, 2022; Stanojlovic et al., 2016). Previous work demonstrated that VEGF overexpression enhances neurogenesis and angiogenesis while altering affective behaviors (Udo et al., 2008), suggesting that VEGF's influence on emotionality is closely tied to its neurogenic and neuroprotective properties. Our results indicate that rTMS likely promotes the release of VEGF, which in turn activates VEGFR‐2 to support neuronal health and synaptic connectivity—effects that are circumvented by receptor blockade. This interpretation is supported by studies linking VEGF signaling to the preservation of neuronal structure and function under conditions of stress or injury (Wasnik et al., 2024; Omole et al., 2025).

Several limitations of the current study should be acknowledged. First, it should be noted that at the current stage, no specific rTMS treatment coil existed for the animal studies, so this might be a non‐specific effect, which is more widespread beyond the mPFC. Consistent with this, our conclusions regarding prefrontal VEGFR‐2 signaling should be interpreted with the understanding that SU5416 was administered systemically and rTMS was delivered to the whole brain; the PFC was the region sampled for analysis, but we cannot exclude additional contributions from other brain regions. Second, the use of systemic administration of SU5416, while effective in inhibiting VEGFR‐2 based on total protein reduction and previously published data (Castañeda‐Cabral et al., 2023), may have off‐target effects or influence peripheral systems that indirectly affect central nervous function. We acknowledge that phosphorylated VEGFR‐2 and downstream signaling pathways (e.g., p‐ERK and p‐Akt) were not assessed in this study, which limits direct evidence of target engagement; however, our behavioral and histological data collectively support functional VEGFR‐2 blockade. Future studies employing conditional knockout models or localized intracerebral infusion of antagonists, along with direct phosphorylation measurements, would help to clarify the specific contribution of prefrontal VEGFR‐2 signaling. Third, although our behavioral tests are well‐validated, they primarily reflect depressive‐ and anxiety‐like behaviors in rodents. We acknowledge the absence of SSCE + SU5416 and CON + SU5416 control groups, which limits our ability to fully exclude rTMS‐independent or baseline effects of chronic VEGFR‐2 blockade, as well as drug × treatment interactions. Therefore, while our data demonstrate that VEGFR‐2 signaling is required for rTMS efficacy under the present experimental conditions, our conclusions regarding causality should be interpreted with caution. Our prior work already confirmed SSCE‐induced deficits (Xu et al., 2024; Liang et al., 2023), so this limitation is noted. Finally, the temporal dynamics of VEGF expression and signaling in response to rTMS under SSCE remain to be fully elucidated. It would be valuable in future work to measure VEGF levels at multiple time points and to examine downstream signaling pathways, such as PI3K/Akt and MAPK/ERK, which are known to be activated by VEGFR‐2 and implicated in neuroplasticity (Feng et al., 2021; Zhang et al., 2021; Deyama et al., 2019). Besides, our study only used male C57BL/6J mice, and the results may not be applicable to females. This is a limitation that should be acknowledged.

## 5 Conclusion

In conclusion, this study demonstrates that VEGFR‐2 represents a promising molecular target through which rTMS exerts therapeutic benefits under simulated spaceflight conditions. Our findings indicate that VEGFR‐2 signaling contributes to the pathogenesis of emotional abnormalities induced by aerospace‐specific environmental stressors, and its activation likely contributes to the neuroprotective and behavioral improvements mediated by rTMS. Targeting the VEGF may thus represent a promising molecular strategy for ameliorating emotional dysregulation in extreme environments (Figure 5). Further investigation is warranted to validate these mechanisms and optimize translational applications for long‐duration space missions.

**FIGURE 5:**
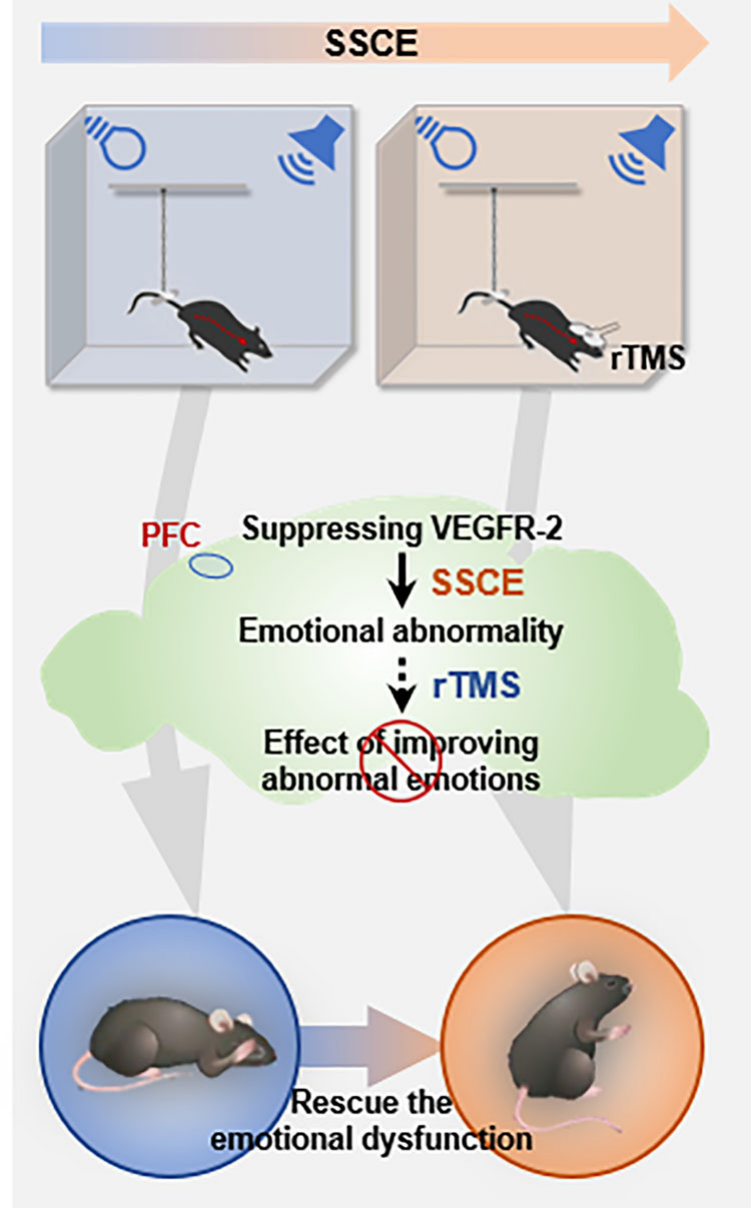
Summary of rTMS‐induced behavioral rescue through prefrontal VEGFR‐2 signaling under simulated spaceflight conditions. Simulated space composite environment (SSCE) exposure triggers emotional dysfunction and social deficits. Therapeutic intervention with repetitive transcranial magnetic stimulation (rTMS) activates VEGFR‐2‐dependent neuroprotection, restoring neuronal integrity and ameliorating depressive‐ and anxiety‐like behaviors. Notably, pharmacological blockade of VEGFR‐2 abolishes these beneficial effects, supporting the involvement of this pathway for rTMS efficacy in extreme environments.

## Author Contributions

**Rong Liang**: funding acquisition, Writing – original draft, Writing – review and editing, methodology, data curation. **Qing Xu**: data curation. **Jing Ren**: data curation, methodology. **Ling Wang**: project administration, resources. **Xinyan Cheng**: data curation. **Fei Zhao**: project administration. **Jing Xu**: project administration. **Jiajia Yang**: project administration, funding acquisition. **Dong Ming**: project administration, resources.

## Funding

This work was supported by grants from the National Natural Science Foundation of China (82371886, 82502266), the China Postdoctoral Science Foundation (2025M782942, 2026T190777), Tianjin Health Research Project (TJWJ2023QN011) and Tianjin Natural Science Foundation (24JCQNJC01080).

## Ethics Statement

All experiments on animals were conducted in accordance with the Declaration of Helsinki. All animal experiments comply with the ARRIVE guidelines. All animal procedures involving animals were conducted in accordance with the approved protocol for the care and use of laboratory animals by the Animal Care and Use Committee of Tianjin University (Animal license number: TJUE: 2023–105). All the authors’ compliance with all relevant ethical regulations.

## Conflicts of Interest

The authors declare no conflicts of interest.

## Data Availability

All analyzed data is included in the article. If anyone needs to access the original data, please contact the corresponding authors (jiajia.yang@tju.edu.cn, richardming@tju.edu.cn).

## References

[brb371799-bib-0001] Abd Al Haleem, E. N. , H. I. Ahmed , and R. N. El‐Naga . 2021. “Lycopene and Chrysin Through Mitigation of Neuroinflammation and Oxidative Stress Exerted Antidepressant Effects in Clonidine‐Induced Depression‐Like Behavior in Rats.” Journal of Dietary Supplements 20, no. 3: 391–410. 10.1080/19390211.2021.1988797 34633271

[brb371799-bib-0002] Baeken, C. , R. De Raedt , A. Bossuyt , et al. 2011. “The Impact of HF‐rTMS Treatment on serotonin2A Receptors in Unipolar Melancholic Depression.” Brain Stimulation 4, no. 2: 104–111. 10.1016/j.brs.2010.09.002. 21511211

[brb371799-bib-0003] Bai, Y.‐W. , Q.‐H. Yang , P.‐J. Chen , and X.‐Q. Wang . 2023. “Repetitive Transcranial Magnetic Stimulation Regulates Neuroinflammation in Neuropathic Pain.” Frontiers in Immunology 14: 1172293. 10.3389/fimmu.2023.1172293. PMC10167032 37180127

[brb371799-bib-0004] Bogdanova, O. V. , S. Kanekar , K. E. D'Anci , and P. F. Renshaw . 2013. “Factors Influencing Behavior in the Forced Swim Test.” Physiology & Behavior 118: 227–239. 10.1016/j.physbeh.2013.05.012. PMC5609482 23685235

[brb371799-bib-0005] Carmeliet, P. , and C. R. de Almodovar . 2013. “VEGF Ligands and Receptors: Implications in Neurodevelopment and Neurodegeneration.” Cellular and Molecular Life Sciences 70, no. 10: 1763–1778. 10.1007/s00018-013-1283-7. PMC11113464 23475071

[brb371799-bib-0006] Castañeda‐Cabral, J. L. , S. A. Orozco‐Suárez , C. Beas‐Zárate , B. F. Fajardo‐Fregoso , M. E. Flores‐Soto , and M. E. Ureña‐Guerrero . 2023. “Inhibition of VEGFR‐2 by SU5416 Increases Neonatally Glutamate‐Induced Neuronal Damage in the Cerebral Motor Cortex and Hippocampus.” Journal of Biochemical and Molecular Toxicology 37, no. 5: e23315. 10.1002/jbt.23315. 36732937

[brb371799-bib-0007] Choi, M. , S. H. Lee , H. L. Chang , and H. Son . 2016. “Hippocampal VEGF Is Necessary for Antidepressant‐Like Behaviors but Not Sufficient for Antidepressant‐Like Effects of Ketamine in Rats.” Biochimica et Biophysica Acta (BBA)—Molecular Basis of Disease 1862, no. 7: 1247–1254. 10.1016/j.bbadis.2016.04.001. 27063455

[brb371799-bib-0008] Clark‐Raymond, A. , and A. Halaris . 2013. “VEGF and Depression: A Comprehensive Assessment of Clinical Data.” Journal of Psychiatric Research 47, no. 8: 1080–1087. 10.1016/j.jpsychires.2013.04.008. 23684549

[brb371799-bib-0009] Cryan, J. F. , C. Mombereau , and A. Vassout . 2005. “The Tail Suspension Test as a Model for Assessing Antidepressant Activity: Review of Pharmacological and Genetic Studies in Mice.” Neuroscience & Biobehavioral Reviews 29, no. 4‐5: 571–625. 10.1016/j.neubiorev.2005.03.009. 15890404

[brb371799-bib-0010] Dang, R. , M. Wang , X. Li , et al. 2022. “Edaravone Ameliorates Depressive and Anxiety‐Like Behaviors via Sirt1/Nrf2/HO‐1/Gpx4 Pathway.” Journal of Neuroinflammation 19, no. 1: 41. 10.1186/s12974-022-02400-6. PMC8822843 35130906

[brb371799-bib-0011] Desai, R. I. , C. L. Limoli , C. E. L. Stark , and S. M. Stark . 2022. “Impact of Spaceflight Stressors on Behavior and Cognition: A Molecular, Neurochemical, and Neurobiological Perspective.” Neuroscience and Biobehavioral Reviews 138: 104676. 10.1016/j.neubiorev.2022.104676. 35461987

[brb371799-bib-0012] Deyama, S. , S. Aoki , R. Sugie , et al. 2023. “Intranasal Administration of Resolvin E1 Produces Antidepressant‐Like Effects via BDNF/VEGF‐mTORC1 Signaling in the Medial Prefrontal Cortex.” Neurotherapeutics 20, no. 2: 484–501. 10.1007/s13311-022-01337-1. PMC10121976 36622634

[brb371799-bib-0013] Deyama, S. , E. Bang , T. Kato , X.‐Y. Li , and R. S. Duman . 2019. “Neurotrophic and Antidepressant Actions of Brain‐Derived Neurotrophic Factor Require Vascular Endothelial Growth Factor.” Biological Psychiatry 86, no. 2: 143–152. 10.1016/j.biopsych.2018.12.014. PMC6597338 30712809

[brb371799-bib-0014] Deyama, S. , E. Bang , E. S. Wohleb , et al. 2019. “Role of Neuronal VEGF Signaling in the Prefrontal Cortex in the Rapid Antidepressant Effects of Ketamine.” American Journal of Psychiatry 176, no. 5: 388–400. 10.1176/appi.ajp.2018.17121368. PMC6494682 30606046

[brb371799-bib-0015] Feng, Y. , X. Li , J. Wang , X. Huang , L. Meng , and J. Huang . 2021. “Pyruvate Kinase M2 (PKM2) Improve Symptoms of Post‐Ischemic Stroke Depression by Activating VEGF to Mediate the MAPK/ERK Pathway.” Brain and Behavior 12, no. 1: e2450. 10.1002/brb3.2450. PMC8785619 34898024

[brb371799-bib-0016] Fukuda, A. M. , L. E. Hindley , J. W. D. Kang , et al. 2020. “Peripheral Vascular Endothelial Growth Factor Changes After Transcranial Magnetic Stimulation in Treatment‐Resistant Depression.” Neuroreport 31, no. 16: 1121–1127. 10.1097/WNR.0000000000001523. PMC7541741 32956213

[brb371799-bib-0017] Gao, J. , J. R. Thonhoff , T. J. Dunn , and P. Wu . 2013. “Lipoic Acid Enhances Survival of Transplanted Neural Stem Cells by Reducing Transplantation‐Associated Injury.” Journal of Neurorestoratology 1: 1–12. 10.2147/JN.S43745. PMC9162033 35663360

[brb371799-bib-0018] Greene, J. , M. Banasr , B. Lee , J. Warner‐Schmidt , and R. S. Duman . 2009. “Vascular Endothelial Growth Factor Signaling Is Required for the Behavioral Actions of Antidepressant Treatment: Pharmacological and Cellular Characterization.” Neuropsychopharmacology 34, no. 11: 2459–2468. 10.1038/npp.2009.68. PMC3694572 19553916

[brb371799-bib-0019] Hecking, I. , L. N. Stegemann , V. Theis , et al. 2022. “Neuroprotective Effects of VEGF in the Enteric Nervous System.” International Journal of Molecular Sciences 23, no. 12: 6756. 10.3390/ijms23126756. PMC9224388 35743202

[brb371799-bib-0020] Hou, Y. , J. Zhao , D. Yang , et al. 2021. “LF‐rTMS Ameliorates Social Dysfunction of FMR1 Mice via Modulating Akt/GSK‐3β Signaling.” Biochemical and Biophysical Research Communications 550: 22–29. 10.1016/j.bbrc.2021.02.086. 33677132

[brb371799-bib-0021] Jin, K. , Y. Zhu , Y. Sun , X. O. Mao , L. Xie , and D. A. Greenberg . 2002. “Vascular Endothelial Growth Factor (VEGF) Stimulates Neurogenesis in Vitro and in Vivo.” Neuroscience 99, no. 18: 11946–11950. 10.1073/pnas.182296499 PMC129374 12181492

[brb371799-bib-0022] Komada, M. , K. Takao , and T. Miyakawa . 2008. “Elevated Plus Maze for Mice.” Journal of Visualized Experiments 00, no. 22: e1088. 10.3791/1088. PMC2762911 19229173

[brb371799-bib-0023] Lange, C. , E. Storkebaum , C. R. de Almodóvar , M. Dewerchin , and P. Carmeliet . 2016. “Vascular Endothelial Growth Factor: A Neurovascular Target in Neurological Diseases.” Nature Reviews Neurology 12, no. 8: 439–454. 10.1038/nrneurol.2016.88. 27364743

[brb371799-bib-0024] Le, Y.‐Z. , B. Xu , A. J. Chucair‐Elliott , H. Zhang , and M. Zhu . 2021. “VEGF Mediates Retinal Müller Cell Viability and Neuroprotection Through BDNF in Diabetes.” Biomolecules 11, no. 5: 712. 10.3390/biom11050712. PMC8150851 34068807

[brb371799-bib-0025] Liang, R. , J. Gao , X. Liu , et al. 2025. “Regulatory Measures for Mitigating Physical and Mental Health Impacts in Aerospace Environment: A Systematic Review.” Life Sciences in Space Research 46: 106–114. 10.1016/j.lssr.2025.04.003. 40774731

[brb371799-bib-0026] Liang, R. , L. Wang , X. L , et al. 2023. “Repetitive Transcranial Magnetic Stimulation Rescues Simulated Space Complex Environment–induced Emotional and Social Impairments by Enhancing Neuronal Excitability in the Medial Prefrontal Cortex.” Cerebral Cortex 33, no. 11: 7148–7162. 10.1093/cercor/bhad027. 36813305

[brb371799-bib-0027] Liang, R. , L. Wang , S. Sun , C. Zheng , J. Yang , and D. Ming . 2022. “Medial Prefrontal Cortex and Hippocampus in Mice Differently Affected by Simulate Microgravity and Social Isolation Associated With the Alternation of Emotional and Cognitive Functions.” Life Sciences in Space Research 33: 21–32. 10.1016/j.lssr.2022.02.001 35491026

[brb371799-bib-0028] Liang, R. , L. Wang , Q. Yang , et al. 2023. “Time‐course Adaptive Changes in Hippocampal Transcriptome and Synaptic Function Induced by Simulated Microgravity Associated With Cognition.” Frontiers in Cellular Neuroscience 17: 1275771. 10.3389/fncel.2023.1275771. PMC10585108 37868195

[brb371799-bib-0029] Liu, M.‐Y. , C.‐Y. Yin , L.‐J. Zhu , et al. 2018. “Sucrose Preference Test for Measurement of Stress‐Induced Anhedonia in Mice.” Nature Protocols 13, no. 7: 1686–1698. 10.1038/s41596-018-0011-z. 29988104

[brb371799-bib-0030] Matsuno, H. , S. Tsuchimine , K. O'Hashi , et al. 2022. “Association Between Vascular Endothelial Growth Factor‐Mediated Blood–Brain Barrier Dysfunction and Stress‐Induced Depression.” Molecular Psychiatry 27, no. 9: 3822–3832. 10.1038/s41380-022-01618-3. 35618888

[brb371799-bib-0031] Morey‐Holton, E. R. , and R. K. Globus . 2002. “Hindlimb Unloading Rodent Model: Technical Aspects.” Journal of Applied Physiology 92, no. 4: 1367–1377. 10.1152/japplphysiol.00969.2001 11895999

[brb371799-bib-0032] Newton, S. S. , N. M. Fournier , and R. S. Duman . 2013. “Vascular Growth Factors in Neuropsychiatry.” Cellular and Molecular Life Sciences 70, no. 10: 1739–1752. 10.1007/s00018-013-1281-9. PMC3951730 23475069

[brb371799-bib-0033] Oluwafemi, F. A. , R. Abdelbaki , J. C.‐Y. Lai , J. G. Mora‐Almanza , and E. M. Afolayan . 2021. “A Review of Astronaut Mental Health in Manned Missions: Potential Interventions for Cognitive and Mental Health Challenges.” Life Sciences in Space Research 28: 26–31. 10.1016/j.lssr.2020.12.002. 33612177

[brb371799-bib-0034] Omole, J. G. , I. A. Okon , G. J. Udom , et al. 2025. “Neurophysiological Mechanisms Underlying Cardiovascular Adaptations to Exercise: A Narrative Review.” Physiological Reports 13, no. 13: e70439. 10.14814/phy2.70439. PMC12238791 40631359

[brb371799-bib-0035] Platz, T. , and J. C. Rothwell . 2010. “Brain Stimulation and Brain Repair—rTMS: From Animal Experiment to Clinical Trials—What Do We Know?” Restorative Neurology and Neuroscience 28, no. 4: 387–398. 10.3233/RNN-2010-0570. 20714064

[brb371799-bib-0036] Pu, J. , Y. Liu , S. Gui , et al. 2020. “Vascular Endothelial Growth Factor in Major Depressive Disorder, Schizophrenia, and Bipolar Disorder: A Network Meta‐Analysis.” Psychiatry Research 292: 113319. 10.1016/j.psychres.2020.113319. 32717712

[brb371799-bib-0037] Ribatti, D. , and D. Guidolin . 2022. “Morphogenesis of Vascular and Neuronal Networks and the Relationships Between Their Remodeling Processes.” Brain Research Bulletin 186: 62–69. 10.1016/j.brainresbull.2022.05.015. 35659566

[brb371799-bib-0038] Romanella, S. M. , G. Sprugnoli , G. Ruffini , K. Seyedmadani , S. Rossi , and E. Santarnecchi . 2020. “Noninvasive Brain Stimulation & Space Exploration: Opportunities and Challenges.” Neuroscience & Biobehavioral Reviews 119: 294–319. 10.1016/j.neubiorev.2020.09.005. PMC8361862 32937115

[brb371799-bib-0039] Rosenstein, J. M. , J. M. Krum , and C. Ruhrberg . 2020. “VEGF in the Nervous System.” Organogenesis 6, no. 2: 107–114. 10.4161/org.6.2.11687. PMC2901814 20885857

[brb371799-bib-0040] Ruiz de Almodovar, C. , D. Lambrechts , M. Mazzone , and P. Carmeliet . 2009. “Role and Therapeutic Potential of VEGF in the Nervous System.” Physiological Reviews 89, no. 2: 607–648. 10.1152/physrev.00031.2008. 19342615

[brb371799-bib-0041] Scott, J. M. , J. Stoudemire , L. Dolan , and M. Downs . 2022. “Leveraging Spaceflight to Advance Cardiovascular Research on Earth.” Circulation Research 130, no. 6: 942–957. 10.1161/CIRCRESAHA.121.319843. PMC8985452 35298305

[brb371799-bib-0042] Shang, X. , B. Xu , Q. Li , B. Zhai , X. Xu , and T. Zhang . 2017. “Neural Oscillations as a Bridge Between Glutamatergic System and Emotional Behaviors in Simulated Microgravity‐Induced Mice.” Behavioural Brain Research 317: 286–291. 10.1016/j.bbr.2016.09.063. 27693609

[brb371799-bib-0043] Sideromenos, S. , C. Lindtner , A. Zambon , O. Horvath , A. Berger , and D. D. Pollak . 2020. “VEGF Treatment Ameliorates Depression‐Like Behavior in Adult Offspring After Maternal Immune Activation.” Cells 9, no. 4: 1048. 10.3390/cells9041048. PMC7226255 32331397

[brb371799-bib-0044] Stanojlovic, M. , X. Pang , Y. Lin , S. Stone , M. Cvetanovic , and W. Lin . 2016. “Inhibition of Vascular Endothelial Growth Factor Receptor 2 Exacerbates Loss of Lower Motor Neurons and Axons During Experimental Autoimmune Encephalomyelitis.” PLoS ONE 11, no. 7: e0160158. 10.1371/journal.pone.0160158 PMC4965096 27466819

[brb371799-bib-0045] Sun, L. , R. Dong , X. Xu , X. Yang , and M. Peng . 2017. “Activation of Cannabinoid Receptor Type 2 Attenuates Surgery‐induced Cognitive Impairment in Mice Through Anti‐Inflammatory Activity.” Journal of Neuroinflammation 14, no. 1: 138. 10.1186/s12974-017-0913-7. PMC5518095 28724382

[brb371799-bib-0046] Sun, P. , S. Wei , X. Wei , et al. 2016. “Anger Emotional Stress Influences VEGF/VEGFR2 and Its Induced PI3K/AKT/mTOR Signaling Pathway.” Neural Plasticity 2016: 1–12. 10.1155/2016/4129015 PMC4769761 27057362

[brb371799-bib-0047] Tian, L. , S.‐S. Sun , L.‐B. Cui , et al. 2020. “Repetitive Transcranial Magnetic Stimulation Elicits Antidepressant‐ and Anxiolytic‐Like Effect via Nuclear Factor‐E2‐Related Factor 2‐Mediated Anti‐Inflammation Mechanism in Rats.” Neuroscience 429: 119–133. 10.1016/j.neuroscience.2019.12.025. 31918011

[brb371799-bib-0048] Tseng, P.‐T. , Y.‐S. Cheng , Y.‐W. Chen , C.‐K. Wu , and P.‐Y. Lin . 2015. “Increased Levels of Vascular Endothelial Growth Factor in Patients With Major Depressive Disorder: A Meta‐Analysis.” European Neuropsychopharmacology 25, no. 10: 1622–1630. 10.1016/j.euroneuro.2015.06.001. 26123242

[brb371799-bib-0049] Udo, H. , Y. Yoshida , T. Kino , et al. 2008. “Enhanced Adult Neurogenesis and Angiogenesis and Altered Affective Behaviors in Mice Overexpressing Vascular Endothelial Growth Factor 120.” The Journal of Neuroscience 28, no. 53: 14522–14536. 10.1523/JNEUROSCI.3673-08.2008. PMC6671242 19118187

[brb371799-bib-0050] Wang, H.‐L. , X.‐H. Xian , Y.‐Y. Wang , et al. 2015. “Chronic High‐Frequency Repetitive Transcranial Magnetic Stimulation Improves Age‐Related Cognitive Impairment in Parallel With Alterations in Neuronal Excitability and the Voltage‐Dependent Ca^2+^ Current in Female Mice.” Neurobiology of Learning and Memory 118: 1–7. 10.1016/j.nlm.2014.11.002. 25451310

[brb371799-bib-0051] Wang, H.‐Y. , D. Crupi , J. Liu , et al. 2011. “Repetitive Transcranial Magnetic Stimulation Enhances BDNF‐TrkB Signaling in Both Brain and Lymphocyte.” Journal of Neuroscience 31, no. 30: 11044–11054. 10.1523/JNEUROSCI.2125-11.2011. PMC3161730 21795553

[brb371799-bib-0052] Wang, L. , Z. Li , C. Tan , et al. 2018. “Physiological Effects of Weightlessness: Countermeasure System Development for a Long‐Term Chinese Manned Spaceflight.” Frontiers of Medicine 13, no. 2: 202–212. 10.1007/s11684-017-0587-7. 29693211

[brb371799-bib-0053] Warner‐Schmidt, J. L. , and R. S. Duman . 2007. “VEGF Is an Essential Mediator of the Neurogenic and Behavioral Actions of Antidepressants.” Proceedings of the National Academy of Sciences 104, no. 11: 4647–4652. 10.1073/pnas.0610282104. PMC1838655 17360578

[brb371799-bib-0054] Wasnik, K. , P. S. Gupta , G. Singh , et al. 2024. “Neurogenic and Angiogenic Poly(*N*‐acryloylglycine)‐*co*‐(acrylamide)‐*co*‐(*N*‐acryloyl‐glutamate) hydrogel: Preconditioning Effect Under Oxidative Stress and Use in Neuroregeneration.” Journal of Materials Chemistry B 12, no. 25: 6221–6241. 10.1039/D4TB00243A. 38835196

[brb371799-bib-0055] Willey, J. S. , S. Aunon‐Chancellor , L. A. Miles , et al. 2022. “αKlotho Decreases After Reduced Weight‐Bearing From Both Spaceflight and Hindlimb Unloading.” NPI Microgravity 8, no. 1: 18. 10.1038/s41526-022-00203-w. PMC9163032 35654945

[brb371799-bib-0056] Xu, Q. , R. Liang , J. Gao , et al. 2024. “rTMS Ameliorates Time‐Varying Depression and Social Behaviors in Stimulated Space Complex Environment Associated With VEGF Signaling.” Life Sciences in Space Research 42: 17–26. 10.1016/j.lssr.2024.04.001. 39067986

[brb371799-bib-0057] Zhang, N. , M. Xing , Y. Wang , H. Tao , and Y. Cheng . 2015. “Repetitive Transcranial Magnetic Stimulation Enhances Spatial Learning and Synaptic Plasticity via the VEGF and BDNF–NMDAR Pathways in a Rat Model of Vascular Dementia.” Neuroscience 311: 284–291. 10.1016/j.neuroscience.2015.10.038. 26518460

[brb371799-bib-0058] Zhang, W. , Y. Wu , H. Chen , D. Yu , J. Zhao , and J. Chen . 2021. “Neuroprotective Effects of SOX5 Against Ischemic Stroke by Regulating VEGF/PI3K/AKT Pathway.” Gene 767: 145148. 10.1016/j.gene.2020.145148. 32949698

[brb371799-bib-0059] Zhao, Q.i , T. Yokozawa , N. Yamabe , K. Tsuneyama , X. Li , and K. Matsumoto . 2010. “Kangen‐Karyu Improves Memory Deficit Caused by Aging Through Normalization of Neuro‐plasticity‐related Signaling System and VEGF System in the Brain.” Journal of Ethnopharmacology 131, no. 2: 377–385. 10.1016/j.jep.2010.07.016. 20637274

